# Clinical Outcomes of COVID-19 and Impact on Disease Course in Patients with Inflammatory Bowel Disease

**DOI:** 10.1155/2021/7591141

**Published:** 2021-11-30

**Authors:** Panu Wetwittayakhlang, Farah Albader, Petra A Golovics, Gustavo Drügg Hahn, Talat Bessissow, Alain Bitton, Waqqas Afif, Gary Wild, Peter L Lakatos

**Affiliations:** ^1^Division of Gastroenterology, McGill University Health Center, Montreal, Canada; ^2^Gastroenterology and Hepatology Unit, Division of Internal Medicine, Faculty of Medicine, Prince of Songkla University, Songkhla, Thailand; ^3^Division of Internal Medicine, McGill University Health Center, Montreal, Canada; ^4^Department of Gastroenterology, Hungarian Defence Forces, Medical Centre, Budapest, Hungary; ^5^Universidade Federal do Rio Grande do Sul, School of Medicine, Graduate Course Sciences in Gastroenterology and Hepatology, Porto Alegre, Brazil; ^6^First Department of Medicine, Semmelweis University, Budapest, Hungary

## Abstract

**Background and Aims:**

The impact of COVID-19 has been of great concern in patients with inflammatory bowel disease (IBD) worldwide, including an increased risk of severe outcomes and/or possible flare of IBD. This study aims to evaluate prevalence, outcomes, the impact of COVID-19 in patients with IBD, and risk factors associated with severe COVID-19 or flare of IBD activity.

**Methods:**

A consecutive cohort of IBD patients who were diagnosed with COVID-19 infection and followed up at the McGill University Health Care Centre was obtained between March 1, 2020, and April 30, 2021. Demographics, comorbidities, IBD (type, treatments, pre- and post-COVID-19 clinical activity, biomarkers, and endoscopic activity), and COVID-19-related outcomes (pneumonia, hospitalization, death, and flare of IBD disease) were analyzed.

**Results:**

A cohort of 3,516 IBD patients was included. 82 patients (2.3%) were diagnosed with COVID-19 infection (median age: 39.0 (IQR 27.8–48.0), 77% with Crohn's disease, 50% were female). The prevalence of COVID-19 infection in IBD patients was significantly lower compared to the general population in Canada and Quebec (3.5% versus 4.3%, *p* < 0.001). Severe COVID-19 occurred in 6 patients (7.3%); 2 patients (2.4%) died. A flare of IBD post-COVID-19 infection was reported in 8 patients (9.8%) within 3 months. Biologic therapy was held during active COVID-19 infection in 37% of patients. Age ≥55 years (odds ratio (OR): 11.1, 95% CI: 1.8–68.0), systemic corticosteroid use (OR: 4.6, 95% CI: 0.7–30.1), active IBD (OR: 3.8, 95% CI: 0.7–20.8), and comorbidity (OR: 4.9, 95% CI: 0.8–28.6) were factors associated with severe COVID-19. After initial infection, 61% of IBD patients received COVID-19 vaccinations.

**Conclusion:**

The prevalence of COVID-19 infection among patients with IBD was lower than that in the general population in Canada. Severe COVID-19, mortality, and flare of IBD were relatively rare, while a large proportion of patients received COVID-19 vaccination. Older age, comorbidities, active IBD disease, and systemic corticosteroid, but not immunosuppressive or biological therapy, were associated with severe COVID-19 infection.

## 1. Introduction

The emergence of coronavirus disease 2019 (COVID-19), caused by the novel severe acute respiratory syndrome coronavirus 2 (SARS-CoV-2), led to a global pandemic of unprecedented magnitude. Patients with advanced age or coexisting illnesses, such as cardiovascular disease, diabetes, chronic lung disease, and immunosuppressive therapy, are at increased risk of COVID-19-related complications and mortality [1–3].

Inflammatory bowel disease (IBD) is a chronic, relapsing, inflammatory disorder of the gastrointestinal tract, including Crohn's disease (CD) and ulcerative colitis (UC). IBD has been associated with an increased risk of infection-related mortality [4, 5] [hazard ratio (HR) = 2.1, 95% confidence interval (CI) = 1.9–2.4] [6]. There is an increasing concern regarding the risk of patients with IBD being infected with the SARS-CoV-2 and at higher risk of severe COVID-19 complications, potentially due to either the consequences of underlying chronic inflammation or the impact of long‐standing exposure to immunosuppressive and/or targeted biological therapies. In addition, SARS-CoV-2 binds to their target cells through angiotensin-converting enzyme 2 (ACE2). The overexpression of ACE2 receptor in patients with IBD might render patients more susceptible to SARS-CoV-2 infection via the gut mucosal barrier and may increase the risk of cytokine release syndrome associated with lung injury and severe outcomes [7–9].

Multiple studies have been published on the impact of COVID-19 in patients with IBD. Importantly, the current data suggest that IBD patients do not seem to be at a higher risk of being infected by SARS-CoV-2 than the general population, with a relative risk of 0.47 (95% CI: 0.18–1.26) [10–12]. However, the mortality rates of COVID-19 in IBD patients show a much wider range in variability (0%–20.0%) [13, 14].

In the international registry database of COVID-19 in patients with IBD (SECURE-IBD), corticosteroids but not antitumor necrosis factor (anti-TNF) were associated with adverse outcomes of the COVID-19 infection (odds ratio (OR): 6.9; 95% CI: 2.3–20.5) [15]. A subsequent study reported that combination therapy with anti-TNF and thiopurine (OR: 4.01, 95% CI: 1.65–9.78) was associated with an increased risk of severe COVID-19 [16]. However, data on whether SARS-CoV-2 infection affects the disease course of IBD and the consequent impact of COVID-19 on IBD therapies are limited.

Therefore, we aimed to investigate the prevalence and outcome of COVID-19 among patients with IBD, risk factors associated with severe COVID-19, disease course of IBD, and a flare IBD after COVID-19 infection. Furthermore, we evaluated the impacts of COVID-19 on health care in IBD patients during the pandemic, adherence to IBD-related treatment, and COVID-19 vaccination.

## 2. Material and Methods

### 2.1. Study Design and Patient Identification

This study comprised a consecutive cohort of patients with IBD (ulcerative colitis and Crohn's disease) that were seen at the Inflammatory Bowel Disease Centre, University Tertiary Referral Center, McGill University Health Care Centre (MUHC), Montreal, Quebec, Canada, between March 1, 2020, and April 30, 2021. Both COVID-19 vaccination and IBD flare data after COVID-19 were recorded until June 30, 2021. This period represents the prevaccination era of the COVID-19 pandemic in Canada, including the first, second, and third waves.

We searched all patients for an available SARS-CoV-2 by polymerase chain reaction (PCR) analysis in the electronic medical record (EMR) of MUHC and in the Dossier Santé Quebec (DSQ) database for each patient individually to ensure that all patients with a possible confirmed COVID-19 infection will be identified. DSQ is a unique provincial patient database in Quebec. The DSQ serves as a value-added digital platform containing demographic, imaging, prescription, and laboratory results performed on patients in the province.

### 2.2. Outcomes

The primary outcomes were the prevalence, outcomes of COVID-19 in IBD patients, and impact of COVID-19 on IBD disease activity. Severe COVID-19 was defined as either COVID-19 pneumonia, requiring hospitalization due to COVID-19-related complications, requiring an intensive care unit (ICU) admission, the use of mechanical ventilation, and/or death. Regarding IBD disease activity, a flare of clinical activity is defined as partial Mayo score ≥4 for UC or HBI score ≥4 for CD.

Our secondary outcomes included (1) prevalence of COVID-19 among the IBD patients compared to the general population, (2) risk factors associated with severe COVID-19, and (3) impact of COVID-19 on IBD therapies and medical care (disruption of biologics, vaccination).

### 2.3. Data Collection

Demographic data, including age, sex, smoking, and comorbidity, were extracted from the electronic patient medical records. COVID-19 PCR tests were collected from the EMR or the DSQ provincial database. In addition, characteristics of IBD [IBD type, Montreal Classification, disease activity at diagnosis of COVID-19 using either the partial mayo (pMayo) score for UC or the Harvey Bradshaw Index (HBI) for CD, Mayo Endoscopic Score for UC, and Simplified Endoscopic activity Score for Crohn's Disease (SES-CD) for CD, as well as baseline biomarkers (C-reactive protein, fecal calprotectin)] and medical therapy for IBD were included.

The following variables regarding confirmed COVID-19 outcomes were collected: date of diagnosis, clinical symptoms, outcomes of COVID-19, medical treatment, holding of IBD medications, and COVID-19 vaccination. Furthermore, data regarding the IBD disease course (symptoms of flare, HBI, pMayo, and post-COVID-19 infection biomarkers) were followed up at least 2 months after diagnosis with COVID-19 infection.

To compare the prevalence rate of COVID-19 in the patients with IBD and the general population, we obtained the number of real-time updated COVID-19 cases from the provincial and national epidemiology database of the government of Canada during the same period (March 1, 2020, to April 30, 2021).

### 2.4. Statistical Analysis

All analyses were performed using SPSS statistical software package version 20. Descriptive statistics were used to describe baseline data and variables related to IBD and COVID-19 outcomes. Continuous variables were expressed as medians and interquartile range (IQR) or mean and standard deviations (SD), depending on the distribution, and compared using *t*-tests. Categorical variables were reported as frequencies with valid percentages and compared using Chi-square tests. For the prevalence analysis, the rate of COVID-19 in the IBD cohort was compared to the general population in Canada and Quebec, using Chi-square tests proportions.

We performed univariable and multivariable logistic regression to estimate the effects of several factors associated with severe COVID-19 and a flare of IBD activity. These factors included age (>55 years versus ≤55 years), gender, BMI (≥30 kg/m^2^ versus <30 kg/m^2^), smoking, comorbidities, IBD characteristics, disease activity, and IBD medications. In the multivariable analyses, we included only the variables with a *pvalue* < 0.2 from the univariable analysis. A *p* value <0.05 was considered statistically significant.

### 2.5. Ethics

The study was approved by the Research Ethics Board (REB) of McGill University Health Care Center (MUHC) under protocol no. 2022-7837. Individual patient-level data were deidentified to maintain confidentiality in all steps of study analysis. This study was conducted in compliance with regulations stated in the 1975 Declaration of Helsinki.

## 3. Results

### 3.1. Baseline Characteristics of IBD Patients with COVID-19 Infection

There were a total of 3,516 IBD patients during the study period between March 1, 2020, and April 30, 2021. 82 (2.3%) patients were tested positive for SARS-CoV-2 PCR analysis [63 patients (77%) had CD, and 19 (23%) patients had UC]. Overall demographic and baseline characteristics of IBD patients with COVID-19 are shown in Table 1.

The median age at COVID-19 diagnosis was 39.0 years (IQR: 27.8–48.0), 50% were female, and 5 (6%) patients were active smokers. At least one comorbidity was reported in 28 (34.1%) patients. Cardiovascular disease and diabetes mellitus were higher in patients with CD compared to UC, 12.7% versus 0% and 7.9% versus 0%, respectively. The most frequent major comorbidities were morbid obesity (17.1%), cardiovascular disease (9.8%), and chronic lung or airway disease (8.5%).

In CD patients, 30 (47.6%) patients had an ileocolonic type, 33.3% had stricturing, and 6.4% had penetrating behavior. Small bowel resection and perianal involvement were found in 23.8% and 19%, respectively. Patients with UC had left-sided or extensive colitis (94.8%) and had moderate severity (68.4%). In both CD and UC, 18 (22.2%) patients had an extraintestinal manifestation of IBD.

Regarding IBD disease activity at the time of COVID-19 diagnosis, most of the patients were in clinical remission. Overall median HBI for CD was 2 (IQR: 0–2), and partial Mayo score for UC was 1 (0–2). Baseline CRP and fecal calprotectin before COVID-19 infection were 4.0 mg/l (IQR: 1.0–8.0) and 165.0 mcg/g (IQR: 36.5–320), respectively. Meanwhile only 18 (22%) patients were clinically active during COVID-19 infection (14 patients with CD and 4 patients with UC). Among these patients with active clinical activity and who had performed an endoscopy to assess endoscopic activity, the median SES-CD score was 9 (IQR: 3–20) in CD, and the Mayo endoscopic score (MES) was 2 (IQR: 2-3) in UC patients.

Most of the IBD patients with COVID-19 infection were treated with biologics (72%), including ustekinumab (35.6%), adalimumab (30.5%), infliximab (18.6%), vedolizumab (10.2%), and other biologics in clinical studies (3.4%) prior to infection. Systemic corticosteroid use within 12 weeks before COVID-19 infection was reported in 9 (11%) patients, with the median dosage of 20 mg/day of prednisone (IQR: 7.5–40.0). Only 3.7% of the patients had thiopurine/mercaptopurine treatment during COVID-19 infection.

### 3.2. Prevalence of COVID-19 in Patients with IBD and the General Population

The prevalence of COVID-19 infection in our IBD cohort (2.33%) was significantly lower than that in the Canadian and Quebec general population during the same period (March 1, 2020, to April 30, 2021). In Canadian population, the total number of COVID-19 cases was 1,219,413 among 35,151,728 individuals [17, 18], with prevalence rate of 3.47% (*p* < 0.001). There were 348,731 infected COVID-19 cases among 8,164,361 individuals in the Quebec population, with a prevalence rate of 4.27% (*p* < 0.001).

### 3.3. Outcome of COVID-19 in Patients with IBD

The majority of the 82 IBD patients with a positive SARS-CoV-2 PCR test were asymptomatic (22.0%) or had mild symptoms (70.7%), including fever, respiratory symptoms, dysosmia, diarrhea, and myalgia. The median post-COVID-19 CRP level was 4.0 mg/L (IQR: 2.0–12.8), and post-COVID-19 fecal calprotectin was 197 mcg/g (IQR: 57.5–653.5) comparable with the pre-COVID-19 infection. Outcomes of COVID-19 and clinical course of IBD are shown in Table 2.

Six patients (7.3%) had severe COVID-19 outcomes and required hospitalization, with a median hospitalization duration of 10.5 days (IQR: 3.8–29.0). Of these, 5 patients (6.1%) had COVID-19-pneumonia, 2 (2.4%) patients required ICU admission for respiratory support by a ventilator, and those two patients died due to multiorgan failure related to COVID-19. The summary data of patients with severe COVID-19 are shown in Table 3.

### 3.4. Disease Course of IBD after COVID-19 Infection

Among IBD patients with COVID-19 infection, 8 patients (9.8%) had an acute flare of IBD disease after COVID-19 infection. The median duration of the flare was 9.5 weeks (IQR: 7.2–12.0). The median CRP was 8.0 mg/l (IQR: 3.0–42.0) and FCAL was 1,088 mcg/g (IQR: 562–2,025) in these patients.

### 3.5. Impact of COVID-19 on Medical Care in Patients with IBD

Among 82 patients with COVID-19 infection during the follow-up period, most patients were adherent to their IBD therapies. However, in the patients receiving biologic therapy, 30 of 59 patients (36.6%) had to hold or had disrupted the biologic use regarding the concern of COVID-19 complications or infusion scheduling disruption, with a median duration of holding biologics of 3 weeks (IQR: 2.0–5.0). None of the biologics-treated patients have discontinued medications after the resolution of COVID-19 symptoms. Moreover, 7 (8.5%) patients were delayed in endoscopy due to the procedure limitation during the pandemic, and 15 (18.3%) patients were lost to follow-up after diagnosis with COVID-19 infection.

### 3.6. Risk Factors Associated with Severe COVID-19 and Flare of IBD

The factors associated with severe COVID-19 outcomes using univariate and multivariate analyses, including age, comorbidities, clinical activity, and concomitant medical treatment for IBD, are shown in Table 4. Univariate analyses showed that increased age >55 years [odds ratio (OR): 11.09, 95% CI: 1.81–68.09, *p*=0.02], systemic corticosteroid use (OR: 4.64, 95% CI: 0.72–30.06, *p*=0.08), clinically active IBD (OR: 3.80, 95% CI: 0.69–20.78, *p*=0.100), and ≥1 comorbidity (OR: 4.86, 95% CI: 0.83–28.6, *p*=0.08) were associated with COVID-19-related complications (hospitalization, pneumonia, and/or death). We used a cut-off at 55 years of age regarding most patients below 60 years of age in our cohort. However, this potential risk factor was not confirmed in multivariable analyses. We identified only older age (>55 years) as an independent risk factor to develop severe COVID-19 (OR: 10.86, 95% CI: 1.64–72.07, *p*=0.013) in the multivariate analyses. We excluded comorbidity from the final multivariate analyses to minimize confounding factors, since it is not an independent variable from increasing age.

However, no association was found between smoking, IBD type, the extension of the disease, or biologics/immunomodulators therapies and the risk of severe COVID-19. In addition, we did an exploratory analysis of the risk factors associated with the flare of IBD activity after COVID-19 infection but did not find any significant factors.

### 3.7. COVID-19 Vaccination in IBD Patients after COVID-19 Infection

At the time of study data collection, 50 of 82 patients (61.0%) had received at least the first COVID-19 vaccine dose after COVID-19 infection: 39 (47.6%) received only one dose of vaccine, and 11 (13.4%) patients received two doses of vaccine after being diagnosed with COVID-19 infection (exclusively mRNA vaccines, Pfizer or Moderna).

## 4. Discussion

This study comprehensively investigated the prevalence, clinical outcomes, impact of COVID-19 on IBD disease course, and predictive factors of developing severe COVID-19 infection in patients with IBD in a large Canadian province. The prevalence of COVID-19 infection was 2.3% among the IBD patients, which was significantly lower than that in the general population in Canada and Quebec, 3.5% and 4.3%, respectively. The COVID-19-related hospitalization and mortality in IBD patients were 7.3% and 2.4%, respectively. We also evaluated the disease course of IBD and disease activity before and after COVID-19 infection. 9.8% of IBD patients had a flare of IBD activity after COVID-19 infection. An active IBD disease activity, systemic corticosteroid use, and comorbidities were likely associated with severe COVID-19 outcomes. Meanwhile older age (>55 years) was the only independent risk factor for developing severe COVID-19. However, immunosuppressive or biological therapies did not find an increased risk of severe COVID-19.

The prevalence of COVID-19 infection among IBD patients in our cohort was lower than that in the Quebec and Canadian general population. A minority of COVID-19 vaccinations occurred until the end of April 2021 in Canada. It can be speculated that the lower prevalence rates can be at least partially explained by more stringent self-isolation of IBD patients, particularly those treated with biologics or immunosuppressants. Of note, Crohn's and Colitis Canada (CCC) [19], the IBD patient organization of Canada, reacted very quickly after the pandemic started, and the developed early stratified self-isolation recommendations for IBD patients. Our prevalence of COVID-19 infection in patients with IBD is numerically comparable with Italian and Spanish cohorts, ranging from 3.0 to 3.5% [13, 20–22]. However, it is important to note that those rates occurred during the first wave of the pandemic, while our study assessed the rates during the first to third waves of the pandemic.

Severe COVID-19 and mortality in patients with IBD were 7.3% and 2.4% in the present study, similar to data from an international registry study (SECURE-IBD) from Brenner et al., which reported that 7% of IBD patients had severe COVID-19 with 3% fatality rate [15]. In addition, in a Swedish population-based cohort, it was found that patients with IBD were more likely to be admitted to hospital for COVID-19 than the general population (adjusted HR = 1.32, 95% CI: 1.12–1.56), but the risk of severe COVID-19 was not higher (adjusted HR = 1.12, 95% CI: 0.85–1.47) [23]. In contrast, severe COVID-19 and mortality rates were higher in some other studies [14, 24]. A nationwide cohort from Netherlands reported IBD patients with COVID-19; 59% needed hospitalization, 20% had severe COVID-19, and the mortality was 13.0% [25]. A recent meta-analysis reported a higher rate of severe COVID-19 and mortality in IBD patients, including 24 studies from December 2019 until July 2020 [10]. The authors reported the pooled proportion of COVID-19 positive IBD patients requiring hospitalization and ICU care was 27.3% and 5.3%, while pooled mortality was 4.3%.

The individual risk factors for severe COVID-19 in patients with IBD in our cohort are also similar to previous studies, such as advanced age and comorbidities [14, 15, 26]. L. Derikx et al. reported ≥1 comorbidity as an independent risk factor for hospitalization (OR: 4.20, 95% CI: 1.58–11.17) [25]. We also identified active IBD disease as an increased risk of COVID-19-related complications, consistent with data from an Italian prospective conservational study, reporting the active disease as significantly associated with COVID-19-related death (OR: 10.25, 95% CI: 2.11–49.73) [27].

Systemic corticosteroid use was the only IBD treatment related to developing severe COVID-19 in our cohort. This result was confirmed by N. Khan et al.; corticosteroid use was independently associated with SARS-CoV-2 infection (HR: 1.60, 95% CI: 1.23 to 2.09, *p*=0.001) and the severe COVID-19 outcomes (HR: 1.90, 95% CI: 1.14–3.17, *p*=0.01) [15, 28, 29]. Of note, these findings potentially represent steroid use as part of treatment in patients with active IBD who are predisposed to severe COVID-19 outcomes. Although most of the COVID-19 patients with IBD were biologic-treated in our cohort, the biologic and immunosuppressive therapies did not increase the risk of severe COVID-19 infection. Several studies confirmed that immunosuppressive and biologic therapies were not associated with an increased risk of severe COVID-19 (OR: 1.73; 95% CI: 0.82–3.63) [26, 27, 30, 31]. Moreover, in a retrospective multicenter study of IBD patients under biologics, non-gut-selective agents were associated with a lower incidence of COVID-19 infection and related symptoms compared with the patients on nonbiologic therapies (7.5% versus 18%, *p* < 0.001). Compared with the general population, IBD patients on biologic therapy are not exposed to a higher risk of COVID-19 [32]. A recent study showed that patients with COVID-19 infection who developed autoantibodies had higher severe COVID-19 outcomes [33, 34]. Therefore, it could be speculated that biologic agents could prevent COVID-19 complications associated with immunological dysregulation in IBD patients. In contrast, the data on medication and severe COVID-19 in IBD patients using the SECURE-IBD registry including 1,439 patients found that a combination therapy of anti-TNF and thiopurines may be associated with an increased risk of COVID-19 adverse outcomes compared with anti-TNF or thiopurine monotherapy [adjusted OR: 4.08, 95% CI: 1.73–9.61] [16].

We also evaluated the clinical and endoscopic activities and biomarkers of the IBD patients before and after COVID-19 infection. Most of the patients (78%) had clinical remission, normal biomarkers, and endoscopic remission before diagnosis with COVID-19 infection. After COVID-19 infection, the majority of patients remained in clinical remission, whereas approximately 10% of the patients had a flare-up of IBD disease, with increased CRP and fecal calprotectin (CRP: 4.0 versus 8.00 mg/l, and fecal calprotectin: 165 versus 1,088 mcg/g) after COVID-19 infection.

COVID-19 pandemic affected the medical care of IBD patients, especially in patients with biological treatment. We found that biologics were held in one-third of the IBD patients with active COVID-19 infection until symptoms of COVID-19 were resolved to avoid potential adverse immunosuppressive effects of biological therapies. However, we did not find an association between holding medications and flare of IBD. In SECURE-IBD data, biologic agents were stopped in 25–40%, and immunomodulators were held in 53% of patients due to COVID-19 infection, but they did not check post-COVID-19 flare frequency [35].

Moreover, our study is one of the first to report COVID-19 vaccination after COVID-19 infection; 61% of IBD patients received at least one vaccine dose in the present cohort. However, data on COVID-19 vaccination in IBD populations with COVID-19 are lacking. A recent study of serological responses to the SARS-CoV-2 vaccine reported that antibody concentrations after a single-dose vaccine were higher in patients with prior SARS-CoV-2 infection [36]. As recommended by the International Organization for the Study of Inflammatory Bowel Disease (IOIBD), IBD medications were not held in our cohort at COVID-19 vaccination [37].

The main strength of our study is that we assessed the prevalence and outcomes of COVID-19 infection in a well-characterized IBD cohort. We were able to verify COVID-19 infection by using the EMRs and also the provincial DSQ database, which is a unique database containing all provincial laboratory records, to ensure reliable rates of COVID-19 infection. In addition, we investigated the IBD disease course, medical care, and vaccinations after COVID-19 infection. Finally, we were able to compare the prevalence of COVID-19 infection in IBD patients with the general population of both Canada and the province of Quebec.

Nonetheless, there are limitations of our study. First, patients with mild symptoms may not have been tested, or some patients (although very rarely) may have traveled outside Canada at the time of COVID-19 infection, and thus some cases may have been missed. Second, due to a low event rate for severe COVID-19 in our cohort, the multivariate analysis to assess risk factors of severe COVID-19 is underpowered. Lastly, our study was a retrospective cohort, and a small number of the patients had no follow-up after the COVID-19 infection.

In conclusion, prevalence, severe outcomes, and mortality of COVID-19 in the present cohort at the McGill IBD Center were low. Older age, comorbidities, active IBD disease, and systemic corticosteroid use, but not immunosuppressive or biological therapy, were associated with severe COVID-19 infection. A large proportion of patients received vaccination against COVID-19 after the COVID-19 infection.

CD; Crohn's disease, UC; ulcerative colitis, HBI; Harvey-Bradshaw Index; SES-CD, Simplified Endoscopic activity Score for Crohn's Disease; NA, not available; UC classification: E1, ulcerative proctitis, E2, left-sided colitis, E3, extensive colitis, S1, mild, S2, moderate, S3, severe; CD classification: L1, ileal CD, L2, colonic CD, L3, ileocolonic CD, L4, upper gastrointestinal CD, B1, nonstricturing, nonpenetrating, B2, structuring, B3, penetrating.

## Figures and Tables

**Table 1:** Baseline characteristics of IBD patients with COVID-19 infection.

Characteristics	Overall (*n* = 82)	CD (*n* = 63)	UC (*n* = 19)
Age (years), median (IQR)	39.0 (27.8–48.0)	38.0 (27.0–47.0)	46.0 (34.0–58.0)
Female, *n* (%)	41 (50.0)	30 (47.6)	11 (57.9)
Body mass index (kg/m^2^), mean (SD)	24.0 (22.0–28.0)	24.0 (22.0–28.0)	23.8 (21.3–29.5)
Active smoking, *n* (%)	5 (6.1)	5 (7.9)	0
Duration of IBD (year), median (IQR)	11.5 (6.0–20.2)	12.0 (7.0–21.0)	11.0 (2.0–20.0)
Concomitant therapy for IBD, *n* (%)			
5-Aminosalicylates	18 (22.0)	7 (11.1)	11 (57.9)
Thiopurines	3 (3.7)	3 (4.8)	0
Sulfasalazine	3 (3.7)	2 (3.2)	1 (5.3)
Systemic corticosteroids	9 (11.0)	4 (6.3)	5 (26.3)
Steroid dose (mg/d), median (IQR)	20.0 (7.5–40.0)	7.5 (5.0–32.5)	20.0 (17.5–45.0)
Methotrexate	1 (1.2)	1 (1.6)	0
*Biologics, n (%)*	59 (72.0)	49 (77.8)	10 (52.6)
Infliximab^a^	11 (18.6)	8 (16.3)	3 (30.0)
Adalimumab	18 (30.5)	16 (32.7)	2 (20.0)
Vedolizumab^a^	6 (10.2)	4 (8.2)	2 (20.0)
Ustekinumab^a^	21 (35.6)	20 (40.8)	1 (10.0)
Tofacitinib^a^	1 (1.7)	0	1 (10.0)
Other biologics (clinical trial study)^a^	2 (3.4)	1 (2.0)	1 (10.0)
Previous expose to biologics	29 (35.4)	27 (42.9)	2 (10.5)
Number of comorbidities, *n* (%)			
None	54 (65.9)	43 (68.3)	11 (55.6)
1	18 (22.0)	12 (19.0)	6 (33.3)
2	7 (8.5)	5 (7.9)	2 (11.1)
3	1 (1.2)	1 (1.6)	0
≥4	2 (2.4)	2 (3.2)	0
Major comorbidity, *n* (%)			
Cardiovascular disease	8 (9.8)	8 (12.7)	0
Chronic lung disease	7 (8.5)	4 (6.3)	3 (15.8)
Diabetes mellitus	5 (6.1)	5 (7.9)	0
Obesity (BMI >30 kg/m^2^)	14 (17.1)	10 (15.9)	4 (21.1)
Immune-mediated diseases	6 (7.3)	4 (6.3)	2 (10.5)
Malignancies	2 (2.4)	1 (1.6)	1 (5.3)
Chronic renal disease	1 (1.2)	1 (1.6)	0
IBD characteristics, *n* (%)			
UC type			
E1/E2/E3	NA	NA	1 (5.3)/9 (47.4)/9 (47.4)
S1/S2/S3	NA	NA	5 (26.3)/13 (68.4)/1 (5.3)
CD type			
L1/L2/L3/L4	NA	12 (19.0)/18 (28.6)/30 (47.6)/3 (4.8)	NA
B1/B2/B3	NA	38 (60.3)/21 (33.3)/4 (6.4)	NA
Previous intestinal resection	15 (18.3)	15 (23.8)	0
Perianal involvement/fistula	12 (14.6)	12 (19.0)	0
Extraintestinal manifestation	18 (22.0)	14 (22.2)	4 (21.1)
IBD disease activity before COVID-19 diagnosis			
HBI score for CD, median (IQR)	NA	2 (0–2)	NA
Partial Mayo score for UC, median (IQR)	NA	NA	1 (0–2)
Patients with endoscopy before COVID-19, *n* (%)	39 (47.6)	28 (44.4)	11 (57.9)
SES-CD for CD (median, IQR)	NA	1 (0–7)	NA
Mayo endoscopic score for UC (median, IQR)	NA	NA	1 (0–2)
Patients with clinical active, n (%)	18 (22.0)	14 (22.2)	4 (21.1)
Patients with endoscopic active, n (%)	13 (15.9)	9 (14.3)	4 (22.1)
SES-CD in active CD, median (IQR)	NA	9 (3–20)	NA
Mayo endoscopic score in active UC, median (IQR)	NA	NA	2 (2–3)
Labs before diagnosis of COVID-19, median (IQR)			
Hemoglobin, g/L	132.5 (122.0–146)	133.5 (122–146.5)	131.5 (119–143)
Albumin, g/L	40.3 (38.0–44.0)	41.0 (37.2–43.5)	42.0 (38.0–45.0)
C-reactive protein, mg/l	4.0 (1.0–8.0)	4.0 (2.0–8.0)	2.0 (0.8–13.5)
Fecal calprotectin, mcg/g	165 (36.5–320)	165.5 (37.8–284.5)	165 (33.5–716)

^a^Percentages were calculated as in patients with biologics use.

**Table 2:** Outcome of COVID-19 in IBD patients, disease course of IBD, and vaccination after COVID-19 infection.

COVID-19 outcomes	Number of patients (%)^a^
Asymptomatic	18 (22.0)
Mild COVID-19 symptoms	58 (70.7)
Hospitalization due to COVID-19/severe COVID-19	6 (7.3)
Duration of hospitalization (day), median (IQR)	10.5 (3.75–29.0)
Recovery day after admission, median (IQR)	12.5 (7.0–25.5)
COVID-19 pneumonia	5 (6.1)
Required mechanical ventilators	2 (2.4)
Required intensive care admission	2 (2.4)
Dead	2 (2.4)
C-reactive protein after COVID-19 (mg/l), median (IQR)	4 (2.0–12.8)
Fecal calprotectin after COVID-19 (mcg/g), median (IQR)	197 (57.5–653.5)
*Flare of IBD disease after COVID-19 infection*	8 (9.8)
Duration after COVID-19 (weeks), median (IQR)	9.5 (7.2–12.0)
C-reactive protein during flare (mg/L), median (IQR)	8.0 (3.0–42.0)
Fecal calprotectin during flare (mcg/g), median (IQR)	1,088.5 (562–2,025)
Treatment care outcomes	
Loss to follow-up	15 (18.3)
Disruption of biologics	30 (36.6)
Duration of holding biologics (week), median (IQR)	3.0 (2.0–5.0)
Number of patients delayed in endoscopy	7 (8.5)
*COVID-19 vaccination*	50 (61.0)
1-dose vaccine	39 (47.6)
2-dose vaccine	11 (13.4)

^a^Data are presented as number (%) unless indicated otherwise. CD: Crohn's disease, UC: ulcerative colitis, NA: not available, IQR: interquartile range.

**Table 3:** Summary data of IBD patients with COVID-19 with severe complications.

No.	Age, sex	IBD classification^a^	Comorbidity	IBD activity	IBD medications	COVID-19 symptoms	COVID-19 complications	Outcome
1	58, M	CD (A3L3S1)	CHF, HTN, DM, AKI, COPD, DVT, morbid obesity	Inactive (HBI 2)	No IBD medication (previous adalimumab)	Fever, dyspnea	Pneumonia, respiratory failure, hospitalization (ICU) for 44 days	Death
2	47, F	CD (A1L3 + L4S2) after resection with ileostomy	None	Inactive (HBI 2)	Infliximab	Fever, dyspnea, diarrhea	Pneumonia, hospitalization (24 days)	Recovery
3	68, F	CD (A3L2S1)	ESRD, IHD, SCV thrombosis	Inactive (HBI 2)	No IBD medication	Fever, dyspnea	Pneumonia, respiratory failure, hospitalization (ICU) for 3 days	Death
4	60, M	CD after colectomy with IPAA (A1L3S2)	Pancreatic cancer, psoriasis	Active (HBI 5)	No IBD medication	Fatigue, diarrhea, abdominal pain	Diarrhea and fever, AKI hospitalization (6 days)	Recovery
5	59, F	UC (A2E2S2)	None	Active (partial Mayo 8)	Adalimumab, prednisone 50 mg/day	Fever, cough, dyspnea	Pneumonia, hospitalization (5 days)	Recovery
6	50, M	UC (A2E2S3)	Morbid obesity	Active (partial Mayo 5, MES 3)	Oral 5-ASA, prednisone 20 mg/day, tofacitinib	Fever, dyspnea	Pneumonia, hospitalization (4 days)	Recovery

^a^IBD classification by Montreal classification. *CD: Crohn's disease, UC: ulcerative colitis, HBI: Harvey-Bradshaw Index, SES-CD: Simplified Endoscopic activity Score for Crohn's Disease, MES: Mayo endoscopic score, AKI: acute kidney injury, COPD: chronic obstructive pulmonary disease, CHF: congestive heart failure, DVT: deep vein thrombosis, DM: diabetes mellitus, ESRD: end-stage renal failure, HT: hypertension, IHD: ischemic heart disease, SCV: subclavian vein, 5-ASA: 5-aminosalicylic acid, ICU: intensive care unit*.

**Table 4:** Predictive factors associated with severe COVID-19 outcome.

Factor variable	Univariate OR (95% CI)	*pvalve*	Multivariate OR (95% CI)	*pvalve*
Age >55 years	11.09 (1.81–68.09)	0.02	10.86 (1.64–72.07)	0.013
Active IBD clinical activity	3.80 (0.69–20.78)	0.10	3.19 (0.41–25.0)	0.269
Systemic corticosteroid	4.64 (0.72–30.06)	0.08	2.08 (0.21–20.78)	0.531
Comorbidity (≥1 major comorbidity)	4.86 (0.83–28.60)	0.08	NA	NA

OR: odds ratio, CI: confidence interval, NA: not available.

## Data Availability

The main data are given in this article. The data are available from the corresponding author upon request.
